# Economic evaluation on dental caries preventive interventions for Australian children using a priority-setting approach

**DOI:** 10.1007/s10198-025-01787-2

**Published:** 2025-04-30

**Authors:** Tan Minh Nguyen, Long Khanh-Dao Le, Hanny Calache, Cathrine Mihalopoulos

**Affiliations:** 1 Health Economics Group, School of Public Health & Preventive Medicine, Faculty of Medicine, Nursing and Health Sciences, Monash University, Level 4, 553 St Kilda Road, VIC 3004 Melbourne, Australia; 2 Deakin Health Economics, Institute for Health Transformation, School of Health and Social Development, Faculty of Health, Deakin University, 221 Burwood Highway, VIC 3125 Burwood, Australia; 3 Dental Health Services Victoria, Level 1, 720 Swanston Street, Carlton, VIC 3053 Australia

**Keywords:** Dental caries, Children, Fluorides, Economic evaluation, Resource allocation

## Abstract

This study aims to economically evaluate three preventive interventions for dental caries among Australian children from low household income. The interventions included: (1) anticipatory guidance provided by oral health therapists via 1a) home visits or 1b) telehealth consultations; (2) school-based dental screening and fluoride varnish program delivered by 2a) dental practitioners or 2b) non-dental health professionals (no screening); and (3) school-based fissure sealant program. The base-case scenario included intervention and dental treatment costs, with six-year (1a and 1b) and two-year (2a, 2b and 3) time horizons. Sensitivity analysis included other healthcare costs (e.g. pulp therapy, extractions, etc.). Additionally, extrapolation modelling extended the 12-year time horizon (all interventions). Probability for cost-effectiveness applied AUD$50,000 per disability-adjusted life year (DALY) averted, and AUD$28,033 per quality-adjusted life year (QALY) gained willingness-to-pay thresholds. Under the base-case scenario, none of the interventions were cost-effective. Cost-effectiveness results were sensitive when including other healthcare costs and the 12-year time horizon. Probability for cost-effectiveness (DALYs averted/QALYs gained) with extrapolation modelling in order of ranking were: (1) 91.5%/94.7% for fluoride varnish, (2) 43.9%/48.6% for dental screening and fluoride varnish, (3) 28.7%/29.8% for anticipatory guidance via telehealth, (4) 17.1%/20.0% for fissure sealant, and (5) 2.2%/2.2% for anticipatory guidance via home visits.

**Supplementary Information:**

The online version contains supplementary material available at 10.1007/s10198-025-01787-2.

## Introduction

Globally, oral diseases affect over 3.5 billion people, with dental caries being the most prevalent health condition (Watt et al. 2019). It has been estimated that there were 0.1 million (95% CI 0.006; 0.3) years lived with disability (YLDs) due to dental caries in the deciduous (baby) teeth and 1.6 million (95% CI 0.7; 3.1) YLDs affecting the permanent (adult) teeth (GBD 2017 Oral Disorders Collaborators et al. 2020). There is increasing inequity from oral diseases. Between 1990 and 2017, reductions in YLDs for all oral disorders were observed for high- and upper-middle-income countries, yet such disorders increased in low- and lower-middle-income countries (GBD 2017 Oral Disorders Collaborators et al. 2020).

In Australia, there was an increase of 5.8% in the age-standardised disability-adjusted life year (DALY) rate for oral disorders between 2003 and 2022 (Australian Institute of Health and Welfare 2022). However, the profile of oral disorders has markedly changed, with a 34.5% decrease for severe tooth loss, while there were increases of 16.7% for dental caries and 40.3% for periodontal disease (Australian Institute of Health and Welfare 2022). High prevalence of oral disease disproportionately impacts people from lower socioeconomic status (Do and Spencer 2016; The Australian Research Centre for Population 2019) and other priority populations including Aboriginal and Torres Strait Islander people, people living in rural and remote areas and people with additional or specialised health care needs (COAG Health Council 2015).

Although dental caries impact populations across the life course, many countries tend to prioritise child populations and people experiencing financial hardship, in their resource allocation decisions within this context (Vernazza et al. 2021; Winkelmann et al. 2022). At a population level in Australia, expenditure for oral healthcare is predominantly incurred via private services with minimal public funding subsidisation, with limited government funding committed to oral healthcare (Nguyen et al. 2023). Dental caries remains one of the most common childhood health conditions and has significant financial burden on families and society (Chen et al. 2021). Therefore, implementing cost-effective dental caries preventive interventions based on rigorous evidence is imperative to improve population oral health.

Economic evaluation on dental caries preventive interventions targeting children have shown mixed results (Nguyen, Tonmukayakul, Le, Calache, et al., 2023). The most common economically evaluated interventions include the application of fissure sealants and fluoride varnish in school-based settings or fixed dental clinics. The variations in study protocol, country context, cost components and outcome measures used make it challenging to identify the best government funding investment decisions. A key limitation, particularly related to economic evaluation of preventive interventions for dental caries, is the commonly used health outcome using the decayed, missing and filled teeth index (DMFT) (Nguyen et al. 2024). The DMFT index measures dental caries experience and is only a ‘proxy’ measure for dental caries. In addition, the diversity of other dental outcome measures used for economic evaluation (Nguyen, Tonmukayakul, Le, Calache, et al., 2023) has meant that making comparisons between oral health interventions are difficult.

The aim of this study is to undertake an economic evaluation of preventive interventions for dental caries targeting Australian children using a population health approach. The three oral health preventive interventions to be evaluated target children from families with low household income and include: (1) anticipatory guidance provided by oral health therapists, (2) school-based fluoride varnish program, and (3) school-based fissure sealant program.

## Methods

For this study, the previously published dental caries economic model from the 20% sugar-sweetened beverages tax intervention was adapted (Nguyen et al. 2023) The present study forms part of the priority-setting study called the Assessing Cost-Effectiveness of Oral Health Preventive Interventions (ACE-Oral Health Prevention) (Nguyen et al. 2023).

Based on our own evidence review on the effectiveness of different preventive interventions for oral health, the three preventive interventions investigated in this study were selected by the Project Steering Group. These dental caries preventive interventions were prioritised using pre-determined selection criteria (Supplementary File, page 1) and were of most relevance to the Australian context. Amongst other factors the criteria included strength of evidence according to the National Health and Medical Research Council evidence hierarchy (Merlin et al. 2009).

This study was reported following the Consolidated Health Economic Evaluation Reporting Standards 2022 (Husereau et al. 2022) (Supplementary File– CHEERS Checklist).

### Effectiveness of dental caries preventive interventions

The clinical effectiveness for the three broad categories of oral health prevention interventions for dental caries relevant to the Australian context were selected and modelled. All interventions were compared against the no intervention comparator (base-case scenario) to reflect the potential impact of these interventions if implemented extensively in the real-world.

Modelling was performed for the appropriate age groups corresponding to the following three interventions.

### Anticipatory guidance provided by oral health therapists

Anticipatory guidance for oral health involves providing oral health education and supporting behaviour change to prevent dental caries (Nation et al. 2023). Anticipatory guidance is the process of providing practical and developmentally appropriate information about children’s health to prepare parents for significant physical, emotional, and psychological milestones (American Academy of Pediatric Dentistry 2022). The modelled intervention includes the provision of oral health education, dietary advice and tooth brushing instructions. The mode of delivery (i.e. community-based, home visits or telehealth) can vary, including the provider of the service (health professional). For anticipatory guidance interventions, effectiveness to prevent dental caries for deciduous teeth was based on the Australian trial provided by oral health therapists via home visits (Intervention 1a) and telehealth (via phone call) (Intervention 1b) (Koh et al. 2015). The intervention was implemented every six months for children starting at 6 months, to age 5 years old (a total of 11 anticipatory guidance consultations).

### School-based fluoride varnish program

Traditionally, fluoride varnish is used in dental settings (clinic-based) as a dental medicine, which consists of a high concentration of fluoride to prevent dental caries and is applied at least every six months (Marinho et al. 2013). The demonstrated effectiveness for dental caries prevention is applicable for both deciduous and permanent teeth, is based on the Cochrane systematic review and meta-analysis study Marinho et al. 2013) and was provided through outreach school-based settings. Fluoride varnish is easy to apply to teeth and has very minimal adverse effects across all age (Mascarenhas 2021).

Regular applications of fluoride varnish can be provided by non-dental health professionals (e.g. medical practitioners, nurse practitioners, Aboriginal and Torres Strait Islander health practitioners, dental assistants, etc.) (Nguyen 2017; Nguyen et al. 2023a; Ummer-Christian et al. 2024). In the United States of America, fluoride varnish is provided by non-dental health professionals under the Medicaid program with a step-down reimbursement fee (Kranz et al. 2022), but the practice is limited in Australia due to existing drugs and poisons regulations and government funding arrangements (Skinner et al. 2020, 2021). For this study, two different modes of delivery were modelled, six-monthly fluoride varnish provided by dental practitioners in primary schools (Intervention 2a), and six-monthly fluoride varnish applied by appropriately trained non-dental health professionals (Intervention 2b), for all school children aged 6–12 years old from low socioeconomic household.

### School-based fissure sealant program

Fissure sealants are a thin protective layer of dental material (the same resin-based restorative material used for white restorations of teeth), which is applied to the deep fissures within the chewing surfaces of the first and second permanent molar teeth. The sealant on the chewing surfaces of the molar teeth prevents the ingression of oral bacteria, food and debris into the fissures of the chewing surfaces of the molar teeth, resulting in prevention of the progression of dental caries on the chewing surfaces of those teeth. In this study, the school-based fissure sealant program was modelled for dental practitioners undertaking outreach school-based settings in primary schools (Intervention 3) for first permanent molars (children aged 6 years and second permanent molars (children aged 12 years). i.e. two cohorts of children aged 6 and 12 years, corresponding to when the first and second permanent molars erupt into the mouth. Intervention effectiveness was derived from the Cochrane systematic review and meta-analysis study (Ahovuo-Saloranta et al. 2017).

### Other intervention components for school-based programs

Typically, school-based dental programs delivered by dental practitioners in Australia include dental screening (Nguyen et al. 2015, 2017). Although there is no evidence for the effectiveness for dental screening to improve oral health outcomes (Arora et al. 2022), the costs for dental screening to identify dental caries risk and referral (first visit only) were included for Intervention 2a and 3 to reflect the real-world scenario. Dental screening is a brief assessment of the teeth, and is differentiated from a dental examination, which includes a thorough evaluation of the teeth, gums and oral cavity. A summary description of the modelled dental caries preventive interventions is reported in Table 1, and key data inputs are reported in Table 2. Detailed parameters that were varied from the dental caries model (Nguyen et al. 2023) can be found in the Supplementary File (Table 1).

**Table 1:** Intervention effectiveness and the cost parameter inputs for the three dental caries preventive intervention targeting children at higher risk in Australia

	Country	Study design	Intervention description		Health provider	Population	Effectiveness	Data source
**Intervention 1a**	Australia	Case-control study	Anticipatory guidance and dental screening provided by oral health therapists during home visits 30-minute consultations (11 visits)		Oral health therapist	6 months	-0.15 mean caries incidence*	Koh et al. 2015
**Intervention 1b**			Anticipatory guidance provided by oral health therapists via telehealth 20-minute consultations (11 visits)		Oral health therapist	6 months	-0.18 mean caries incidence*	
**Intervention 2a**	International	Cochrane systematic review and meta-analysis	Six-monthly fluoride varnish (four visits)		Dental practitioner	6–12 years	0.37 preventive fraction for dmft	Marinho et al. 2013
**Intervention 2b**					Non-dental health professionals	6–12 years	0.43 preventive fraction for DMFT	
**Intervention 3**			Fissure sealant of first or second permanent molars (one visit)		Dental practitioner	6 and 12 years	0.12 odds ratio caries incidence	Ahovuo-Saloranta et al. 2017

dmft/DMFT = decayed, missing and filled teeth (lower case: deciduous dentition / upper case: permanent dentition).

*Clinical effectiveness was based on crude mean value difference from the standard care comparator. Given the authors did not perform statistical analysis for the effectiveness difference, intervention effectiveness was derived using normal distribution parameters generated in the TreeAge Pro 2023 using trial data (Koh et al. 2015).

**Table 2:** Key data inputs for the three dental caries preventive interventions modelled in this study

Data inputs	Intervention 1a	Intervention 1b	Intervention 2a	Intervention 2b	Intervention 3
^Target child population from low household income	45,677 (of 303,407 aged < 1 year old)		333,651 (of 2,236,730 aged 6–12 years old)		94,025 (of 638,445 children aged 6 and 12 years old)
Start age	6 months		6–12 years		6 and 12 years
Time horizon	6 years		2 years		
Intervention effectiveness decay	Intervention effectiveness applied at 100% for all cycles				
Type of model	Closed cohort Markov model				
^+^Intervention mean costs	First year - $1.6 M Subsequent years - $3.2 M	First year - $0.7 M Subsequent years - $1.5 M	First year - $34.6 M Subsequent years - $24.6 M	First year - $18.9 M Subsequent years - $18.9 M	First year - $21.2 M Subsequent years - $0 M
^+^Healthcare costs included	Dental check-up and restorations (AUD$)				
**Sensitivity analysis**					
^+^Other healthcare costs included	*Potentially preventable hospitalisation (D40Z), ^~^dental general anaesthesia and yearly other healthcare costs relevant to dental caries (Supplementary File, page 4)				
**Extrapolation modelling**					
Extended time horizon	12 years				

Intervention 1a: anticipatory guidance provided by oral health therapist via home visits; Intervention 1b: anticipatory guidance provided by oral health therapist via telehealth; Intervention 2a: school-based fluoride varnish program provided by dental practitioners; Intervention 2b: school-based fluoride varnish program provided by non-dental health professionals; Intervention 3: school-based fissure sealant program provided by dental practitioners; ^Data from the Australian Bureau of Statistics (2022); ^+^2020 Australian dollars (AUD$); D40Z: Dental Extractions and Restorations classification by the Australian Refined Diagnosis Related Groups (AR-DRG) Version 10 (Independent Health and Aged Care Pricing Authority 2021); ^~^trial data costs only applied to children < 7 years old (Koh et al. 2015)

### Population

Single age cohorts relevant to the interventions (Table 2) were developed and modelled using TreeAge Pro 2023 with one-year cycle lengths (TreeAge Software, LLC.) for the 2020 Australian population (Australian Bureau of Statistics 2022a). Australian background mortality according to age and sex was incorporated using data from the 2019 Global Burden of Disease (GBD) study (Institute for Health Metrics and Evaluation 2021).

The three preventive interventions for dental caries modelled in this study, includes the child population using the 2021 Australian Census dataset (household income < AUD$60,000/year) (Australian Bureau of Statistics 2022b), which corresponded to the higher prevalence of dental caries among children from low household income (Do and Spencer 2016). Modelled interventions were compared against the ‘status quo’ scenario (i.e. no intervention).

### Time horizon

Two separate time horizons in the base case (Table 2) were selected to reflect the duration on the evidence of effectiveness for the dental caries preventive interventions: (1) six years for anticipatory guidance in early childhood delivered by oral health therapists (Koh et al. 2015) and (2) two years for school-based dental screening with fluoride varnish (Marinho et al. 2013) and school-based fissure sealant program (Ahovuo-Saloranta et al. 2017).

### Health benefit modelling

The research expands on the existing economic evaluation evidence on dental caries prevention by using generic health outcomes as measured using decayed teeth prevented (DT prevented) disability-adjusted life years (DALYs) and quality-adjusted life years (QALYs). Although methodologies to economically model dental caries interventions using DALYs are well established, the use of QALY outcomes in this context are still emerging (Nguyen, Rogers, et al., 2023). QALYs are however the preferred metric to use in many national health technology assessment agencies (National Institute for Health and Care Excellence 2013) including Australian health technology assessment agencies (Commonwealth of Australia 2024).

Modelled health states included dental caries free, dental caries, edentulism (complete tooth loss) and dead (Nguyen et al. 2023). Although edentulism among children is rare, this health state is retained for modelling consistency (Nguyen et al. 2023). Any impacts from edentulism were excluded because edentulism incidence only accrue for age 20 + years (GBD 2019 Diseases and Injuries Collaborators 2020). In order to determine DALYs, disability weights were assigned for dental caries as per the 2019 GBD study (0.010; SD 0.004) (GBD 2019 Diseases and Injuries Collaborators 2020).

Based on the 2019 GBD study, the Australian incidence rate for dental caries for the general population were used (Institute for Health Metrics and Evaluation 2021). Dental caries incidence is derived from the mean number of teeth affected by dental caries (decayed), previously extracted or filled (restored) in the population, also known as DMFT (Lewis 1996). i.e. an increase of 0.10 DMFT represents 10 new cases (individuals) developing dental caries per year.

Dental caries incidence was adjusted based on the higher prevalence of dental caries among children from low household income (Do and Spencer 2016). Differences in dental caries incidence for deciduous and permanent teeth were included for cohort specific mixed dentition for age 6–14 years (Nelson and Wheeler 2020). For each new case of dental caries, there was on average 1.64 (SD 1.262) decayed teeth (Hummel et al. 2019).

Quality of life utility gain, required to calculate QALYs, to prevent dental caries was derived from the Australian trial on anticipatory guidance provided by oral health therapists, which was previously economically evaluated (Koh et al. 2015). The difference between healthy (score of 1) and the baseline health utility for dental caries of 0.9 (SD 0.120) was used (Koh et al. 2015). Health utility was measured using the modified Child Health Utility 9 Dimensions paediatric quality of life multi-attribute instrument (Stevens 2012), with Australian utility population norm being less than 1 (Catchpool et al. 2019; Le et al. 2021). To reflect a more realistic health utility gain as applied in the Australian trial (Koh et al. 2015), we considered a utility gain of 0.1 was optimistic and halved this when the ‘dental caries’ health state moved to ‘healthy’.

### Perspective

The Australian government healthcare perspective (intervention and dental caries treatment costs) was adopted in this study given these interventions are reliant on the public healthcare system for implementation.

### Intervention costs

All costs were adjusted to 2020 (AUD$) prices (Reserve Bank of Australia 2024) based on the consumer price index, consistent with the same reference year for the 20% sugar-sweetened beverages tax intervention to enable comparability between studies consistency (Nguyen et al. 2023).

Table 2 and the Supplementary File (Table 1) reports the intervention costs and all resource inputs, including trial data costings (Koh et al. 2015) for interventions 1a and 1b, or relevant government dental fee-for-service items for children and adults (Department of Veterans’ Affairs 2020) for interventions 2a, 2b and 3.

Anticipatory guidance provided by oral health therapists via home visits (Intervention 1a) included five visits of AUD$23.33 consultation costs and AUD$12.34 (SD 0.92) travel costs (Koh et al. 2015).

Anticipatory guidance provided by oral health therapists via telehealth (Intervention 1b) included 11 visits of AUD$16.04 consultation costs (Koh et al. 2015).

The school-based fluoride varnish program provided by dental practitioners (Intervention 2a) included AUD$29.25 dental screening cost (first visit only) and AUD$36.80 for six-monthly fluoride varnish applications (a total of four visits).

The school-based fluoride varnish program provided by non-dental health professionals practitioners (Intervention 1b) included six-monthly fluoride varnish applications. A 76.9% step-down reimbursement rates of fluoride varnish were applied for non-dental health professionals (Virginia Health Catalyst 2021). Travel costs were excluded due to unknown cost estimations per child, and consistent with previous economic evaluation of a school-based dental program (Nguyen et al. 2017).

The school-based fissure sealant program provided by dental practitioners (Intervention 3) included AUD$29.25 dental screening cost (first visit only) and the cost to apply fissure sealants at AUD$49.00 each to all four first or second permanent molars completed in one visit (four teeth at age six and age 12 years).

### Healthcare costs

The cost consequences due to dental caries included the costs of a dental check-up costing AUD$54.69 (SD 3.19) and the cost of a dental restoration per tooth of AUD$189.61 (SD 17.95) (Nguyen et al. 2023).

### Discount rate

A discount rate of 3% was applied for both costs and outcome measures, consistent with the previous published dental caries model (Nguyen et al. 2023).

### Willingness-to-pay threshold

For the Australian context, the willingness-to-pay (WTP) threshold for dental outcomes is yet to be elicited. Thus, cost-effectiveness was not assessed for preventing decayed teeth. For generic health outcome measures, the WTP of AUD$50,000 per DALY averted (Ciketic et al. 2010; Cobiac and Vos 2012) and AUD$28,033 per QALY gained (Edney et al. 2018) were used.

### Uncertainty analysis

Monte Carlo simulation with 2,000 cycles using within cycle correction was performed in TreeAge Pro 2023 to conduct probabilistic sensitivity analysis. Summary statistics and the incremental cost-effectiveness ratio (ICER) were analysed using Stata 18 BE (StataCorp). Key parameters varied in the model structure were the start age, number of children in the single age cohorts, intervention effectiveness and intervention cost parameters (Supplementary File, Table 1).

### Sensitivity analysis

#### Other healthcare costs

Given the assumptions used in the modelling, an alternative scenario was evaluated that also included other selected healthcare costs relevant to prevent and treat dental caries (e.g. pulp therapy, extractions, etc.) (Supplementary File, page 4). Costs were weighted according to 15% of dental services provided in the public and 85% of dental services provided in the private sector (Tennant and Kruger 2014).

In addition for Intervention 1a and 1b only, the alternative scenario analysis included the costs for potentially preventable hospitalisations due to dental caries based on the trial data (Koh et al. 2015). These costs were estimated according to the Australian Refined Diagnosis Related Groups classification system code D40Z - Dental extraction and restorations (Independent Health and Aged Care Pricing Authority 2021) and the cost for dental general anaesthesia (Koh et al. 2015). Only children aged < 7 years old and those experiencing toothache accrued the costs for potentially preventable hospitalisations due to dental caries and the costs for dental general anaesthesia.

#### Extrapolation modelling

To estimate the economic impact in the longer term, all interventions were modelled with a 12-year time horizon, inclusive of other healthcare costs. The 12-year time horizon was selected to reflect the health impacts according to the ages in which children attended primary and secondary school. This meant that all children in Intervention 1a and 1b would have completed primary school (extended by six years), and for Intervention 2a, 2b and 3, all children would have completed secondary school (extended by 10 years). Intervention effectiveness was assumed to be the same when extrapolated to 12 years, assuming the intervention costs remained fixed to six years for anticipatory guidance provided by oral health therapists (Intervention 1a and 1b; intervention effectiveness only applied to deciduous teeth), and two years for the school-based interventions (Intervention 2a, 2b and 3). A 0% intervention effectiveness decay rate was applied in the absence of existing evidence to inform it.

#### Model validation

Details for the model validation process is described in the previously published dental caries model (Nguyen et al. 2023) and that is the basis for this study, which followed the ISPOR-SMDM Modeling Good Research Practice guidelines (Eddy et al. 2012).

#### Implementation considerations

Following previous methodology, other implementation considerations were rated by the members of the PSG according to the pre-defined criteria (Nguyen et al. 2023), which were informed by the relevant literature, wherever possible.

## Results

### Base-Case scenario

For anticipatory guidance via home visits, the cost difference was $AUD$23.0 million (SE < 0.1 million), which corresponds to 7,649 DT-prevented (SE 93), 0.4 DALYs averted (SE < 0.1) and 2.4 QALYs gained (SE 0.1). Anticipatory guidance via telehealth had a cost difference of AUD$18.5 million (SE < 0.1 million) and yielded 8,927 DT-prevented (SE (113), 0.5 DALYs averted (SE < 0.1) and 2.8 QALYs gained (SE < 0.1).

The dental screening and fluoride varnish program had a cost difference of AUD$57.5 million (SE 0.2 million). The fluoride varnish program provided by non-dental health professionals yielded a cost difference of AUD$34.7 million (SE 0.2 million). Both fluoride varnish interventions generated 65,198 DT-prevented (SE 728), 5.0 DALYs averted (SE < 0.1) and 28.7 QALYs gained (SE 0.6).

The dental screening and fissure sealant program had a cost difference of AUD$10.5 million.

(SE < 0.1 million) and resulted in 1,860 DT-prevented (SE 20), 0.1 DALYs averted (SE < 0.1) and 0.7 QALYs averted (SE < 0.1) compared to no intervention.

The healthcare cost difference, effectiveness difference and the ICER values are reported in Table 3 (undiscounted results reported in Supplementary File, Table 2), which included the base-case scenario and sensitivity analysis. All modelled interventions were not cost-effective (0% probability) under the base-case scenario.

**Table 3:** The healthcare cost difference, effectiveness differences, the ICER values and the probability for cost-effectiveness for the three dental caries preventive intervention targeting children at higher risk in Australia (discounted)

Base-Case Scenario	∆ Healthcare Costs* AUD$ (SE)	∆ DT Effectiveness* (SE)	∆ DALY Averted* (SE)	∆ QALY Effectiveness* (SE)	Incremental Cost-Effectiveness Ratio				
					AUD$ / DT Prevented	AUD$ / DALY Averted	CE	AUD$ / QALY Gained	CE
Home Visits	23.0 million (< 0.1 million)	7,649 (93)	0.4 (< 0.1)	2.4 (0.1)	3,010	54.5 × 10^6^	0%	9.5 × 10^6^	0%
Telehealth	18.5 million (< 0.1 million)	8,927 (113)	0.5 (< 0.1)	2.8 (< 0.1)	2,067	37.2 × 10^6^	0%	6.6 × 10^6^	0%
Dental screening and fluoride varnish	57.5 million (0.2 million)	65,198 (728)	5.0 (< 0.1)	28.7 (0.6)	882	11.4 × 10^6^	0%	2.0 × 10^6^	0%
Fluoride varnish (non-dental health professionals)	34.7 million (0.2 million)				532	6.9 × 10^6^	0%	1.2 × 10^6^	0%
Dental screening and fissure sealant	10.5 million (< 0.1 million)	1,860 (20)	0.1 (< 0.1)	0.7 (< 0.1)	5,651	82.2 × 10^6^	0%	14.4 × 10^6^	0%
**Sensitivity Analysis^** **Including Other Healthcare Costs**									
Home Visits	12.8 million (< 0.1 million)	7,649 (93)	0.4 (< 0.1)	2.4 (0.1)	1,680	30.4 × 10^6^	0.1%	5.3 × 10^6^	0.1%
Telehealth	6.5 million (< 0.1 million)	8,927 (113)	0.5 (< 0.1)	2.8 (< 0.1)	733	13.2 × 10^6^	7.2%	2.3 × 10^6^	7.3%
Dental screening and fluoride varnish	50.7 million (0.2 million)	65,198 (728)	5.0 (< 0.1)	28.7 (0.6)	778	10.1 × 10^6^	0%	1.8 × 10^6^	0%
Fluoride varnish (non-dental health professionals)	27.9 million (0.2 million)				428	5.6 × 10^6^	0.1%	1.0 × 10^6^	0.1%
Dental screening and fissure sealant	10.3 million (< 0.1 million)	1,860 (20)	0.1 (< 0.1)	0.7 (< 0.1)	5,544	80.6 × 10^6^	0%	14.1 × 10^6^	0%
**Sensitivity Analysis^** **12 Year Time Horizon**									
Home Visits	9.6 million (< 0.1 million)	18,303 (221)	1.1 (< 0.1)	6.3 (0.1)	523	8.7 × 10^6^	2.2%	1.5 × 10^6^	2.2%
Telehealth	2.8 million (< 0.1 million)	21,129 (265)	1.3 (< 0.1)	7.2 (0.1)	132	2.2 × 10^6^	28.7%	0.4 × 10^6^	29.8%
Dental screening and fluoride varnish	< 0.1 million (0.6 million)	296,834 (3,316)	39.5 (0.3)	225.6 (4.4)	~ 0	1,988	43.9%	348	48.6%
Fluoride varnish (non-dental health professionals)	-25.0 million (0.6 million)				-84^#^	-663,657^#^	91.5%	-110,984^#^	94.7%
Dental screening and fissure sealant	3.0 million (< 0.1 million)	28,942 (323)	4.1 (< 0.1)	23.3 (0.5)	104	737,176	17.1%	129,035	20.0%

^ includes costs of potentially preventable dental hospitalisation and general anaesthesia for children < 7 years old and other healthcare costs related to dental caries;

AUD$ = 2020 Australian dollars; ∆ = change; *3% discount rate was applied; DT = decayed teeth; DALY = disability-adjusted life years; QALY = quality-adjusted life years; CE = probability intervention is cost-effective (AUD$50,000 per DALY averted and AUD$28,033 per QALY gained); ~ approximately; ^#^ dominant.

### Sensitivity analysis

#### Other healthcare costs

The probability for cost-effectiveness was not sensitive when including other healthcare costs related to dental caries for both DALY outcomes (0-7.2%) and QALY outcomes (0-7.3%) (Table 3).

#### Extrapolation modelling

When the model was extended to the 12-year time horizon, anticipatory guidance via home visits remained not cost-effective (2.2% for DALYs averted and 2.2% QALYs gained). The probability for cost-effectiveness increased for anticipatory guidance via telehealth at 28.7% for DALYs averted and 29.8% for QALYs gained.

The dental screening and fluoride varnish program had increased probability for cost-effectiveness (approximately cost-neutral; 43.9% for DALYs averted and 48.6% for QALYs averted). Evaluation with the 12-year time horizon demonstrated the fluoride varnish program provided by non-dental health professionals was cost-effective (dominant; 91.5% for DALYs averted and 94.7% for QALYs gained).

The dental screening and fissure sealant program had increased probability for cost-effectiveness at 17.1% for DALYs averted and 20.0% for QALYs gained.

#### Implementation considerations

The rating for other implementation considerations is reported in the Supplementary File (Table 3). School-based interventions were considered more favourable when compared to anticipatory guidance interventions. The utilisation of oral health therapists to deliver anticipatory guidance via home visits or telehealth were rated as having low feasibility and sustainability, largely due to constrained workforce supply and state/territory funding mechanism that typically is not flexible to deliver outreach-based interventions.

## Discussion

The findings from this study demonstrates that the three dental caries preventive interventions targeting children from low household income were not cost-effective for the base-case scenario. The ranking for the most to least cost-effective interventions according to the base-case scenario were: (1) school-based fluoride varnish program provided by non-dental health professionals, (2) school-based dental screening and fluoride varnish program, (3) anticipatory guidance via telehealth, (4) anticipatory guidance via home visits, and (5) school-based dental screening and fissure sealant program. The economic evaluation results were sensitive only under extrapolation modelling using the optimistic 12-year time horizon.

The original economic evaluation study on anticipatory guidance via home visits and telehealth provided by oral health therapists, showed they were cost-effective interventions (Koh et al. 2015). This differed from our study, possibly due to two key factors. Firstly, there is potentially selection bias where the chosen comparator were children who were users of public dental services. i.e. children accessing public dental services have more severe levels of dental caries, higher dental caries prevalence (Do and Spencer 2016), and consequently would incur higher costs. Secondly, we used a more conservative value for 0.050 utility gains for each dental caries case prevented within one year because this reflects the unrealistic likelihood a child would be in ‘perfect’ health if they remained free of dental caries (Le et al. 2021). This appears reasonable given the mean QALYs gained for school children was 0.05 (95% -0.01; 0.12) in an economic evaluation of community water fluoridation in Cumbria, UK (Whittaker et al. 2024).

The rankings for the school-based dental caries prevention interventions would appear logical given that the intervention effectiveness for fluoride varnish is applicable to the entire dentition, compared to fissure sealants, which only have a preventive effect for the chewing surfaces of permanent molar teeth. Previous economic evaluation of fissure sealant and fluoride varnish interventions has had mixed cost-effectiveness results (Nguyen, Tonmukayakul, Le, Calache, et al., 2023). It should be noted that it is ethically problematic to explicitly target individual children from low household income for school-based interventions given the diversity of student enrolment. School-based oral health interventions would be implemented by selecting schools based on the most disadvantaged score using the Index of Community Socio-Educational Advantage (ICSEA) classification. The ICSEA provides a relative description of school according to the indication of the socio-educational backgrounds of students (Australian Curriculum, Assessment and Reporting Authority 2013).

Critically, our study results differed from the economic evaluation of the school-based fissure sealant program for the United States context that reported the intervention was cost-effective (cost-saving) (Griffin et al. 2016). Both this study (Griffin et al. 2016) and our study applied the same intervention effectiveness for fissure sealants from the Cochrane review (Ahovuo-Saloranta et al. 2017) and the use of DALYs averted for outcomes. Critically though, the US modelled study applied DALYs averted for 72.1% of children who qualified for Medicaid (a proxy measure for low household income) and had symptomatic dental caries for the entire year (Griffin et al. 2016). This is likely to overestimate health benefits when compared to our dental caries model, which applied DALYs averted for symptomatic dental caries for a shorter duration (28 days) and for a lower proportion of children experiencing symptoms of pain which ranged from 8.4 to 21.0% (Do and Spencer 2016; Nguyen et al. 2023b).

Our DALY estimates are more reasonable given symptoms due to dental caries ranges from 9.0 to 52.1% used in the 2019 GBD study for deciduous and permanent teeth (GBD 2019 Diseases and Injuries Collaborators 2020). Additionally, our study more realistically accounted for the specificity of health benefits for fissure sealants by applying the intervention effectiveness only at the occlusal tooth surface-level (and not the whole tooth) with the two-year time horizon compared to the four-year (base-case) and nine-year (sensitivity analysis) time horizon in the US study (Griffin et al. 2016). i.e. we assumed dental caries incidence for in the school-based fissure sealant program is only attributed to dental caries developing on the occlusal surfaces of the first and second permanent molars.

With the base-case scenario, the fluoride varnish interventions we modelled were not cost-effective, which is in contrast to the Australian economic evaluation of six-monthly fluoride varnish applied for adolescents (Nguyen et al. 2020). A key difference is the choice for the 70-year time horizon with no intervention effectiveness decay, and how the health outcome was valued. The previous study derived QALYs gained as the mean utility gain of eight molars as measured in quality-adjusted tooth years (Nguyen et al. 2020), which is inconsistent with other approaches in valuation of QALYs gained in the literature (Nguyen, Tonmukayakul, Le, Calache, et al., 2023). International studies on school-based fluoride varnish interventions have also reported mixed-evidence for cost-effectiveness (Nguyen, Tonmukayakul, Le, Calache, et al., 2023). Methodological concerns were identified. The choice for the usual care comparator in the Canadian study (Norrie and Pharand 2020) is likely to inflate healthcare costs for the comparison group (i.e. greater prevalence and severity of dental caries) and bias the cost-effectiveness result (cost-saving). The UK threshold analysis study valued QALYs using utilities related to ear infections requiring hospitalisation (acute otitis media) and a longer duration of 84 days with disability experience (Kay 2018). Our study demonstrated that non-dental health professionals are preferred for implementing school-based fluoride varnish interventions, largely due to their lower cost. Whilst our modelled fluoride varnish interventions were shown to be not cost-effective within two years, they may be in the longer term. Further research is warranted to confirm whether fluoride varnish interventions are effective and cost-effective (≥ 12-year time horizon).

### Implications for policy and practice

The assessment for implementation considerations of the five oral health preventive interventions investigated in this study were generally more favourable for school-based interventions, which are currently implemented under various models by the Australian states and territory programs (Lara Mayze 2015; Tasmanian Department of Health 2021; Victorian Department of Health 2024). Low ratings were given by the Project Steering Group on feasibility and sustainability for anticipatory guidance provided by oral health therapists either via home visits or telehealth consultations. The low rating reflects the existing funding mechanism of the state and territory programs, which are funded to provide service delivery rather than outreach population oral health interventions. The long-term effectiveness of anticipatory guidance provided by oral health therapists either via home visits or telehealth consultations showed negligible differences when compared to each other (Harrison-Barry et al. 2022) and suggests the least costly intervention to implement (i.e. telehealth) is preferable.

In Australia, preventive health assessments are commonly delivered by maternity and child.

health services in the early years (Schmied et al. 2011). Therefore, it seems logical to train registered non-dental health professionals to implement anticipatory guidance interventions that include oral health, noting it was cost-effective with the base-case scenario. Anticipatory guidance interventions for young children can be augmented with the regular application of fluoride varnish as demonstrated in previous trials among Aboriginal communities with demonstrated significant difference for dental caries prevention (Jamieson et al. 2018, 2019; Slade et al. 2011). The systematic review on economic evaluation on fluoride varnish application in preschoolers did not find ‘convincing evidence’ that the intervention is cost-effective due to methodological issues or restricted to specific settings limitations (Dhyppolito et al. 2023). The review is consistent with the previous systematic review critiquing methodological limitations, making it difficult to interpret their credentials for cost-effectiveness (Nguyen, Tonmukayakul, Le, Calache, et al., 2023).

Our study provides important insights into the relative cost-effectiveness of different dental caries preventive interventions for children. A similar Chilean study showed that dental sealant interventions were more costly and more effective (Mariño et al. 2012), but it could not be assessed for value against a willingness to pay as it did not express outcomes as DALYs or QALYs. However, cost-effective interventions included community-based interventions such as water fluoridation and salt fluoridation, and school-based milk fluoridation and fluoridated mouthrinses, as they were cost-saving and more effective (called dominant interventions) (Mariño et al. 2012). Although fluoride varnish was not investigated in the Chilean study, its clinical effectiveness to prevent dental caries is comparable to fluoride mouthrinses (Marinho et al. 2004; Twetman and Keller 2016). Thus, our study builds on existing economic evaluation methodologies of dental caries preventive interventions, whereby probably for cost-effectiveness can be highly sensitive to longer time horizons.

### Key limitations

Given our study was modelled-based, its results should be interpreted with caution. The main limitation is that our economic evaluation approach relied on intervention effectiveness for dental caries prevention based on differences in dmft/DMFT, which does not accurately measure dental caries (Nguyen et al. 2024). i.e. dmft/DMFT specifically measures dental caries experience. However, we have reported costs per DT prevented as a way to make comparisons between oral health interventions.

A valid way to convert health benefits using dmft/DMFT to quality of life remains challenging. The use of QALYs, which was derived from the Child Health Utility 9D instrument (Stevens 2012) as applied in our study, is likely not sensitive enough to measure dental caries as demonstrated in previous research (Foster Page et al. 2015; Rogers et al. 2022; Weerasuriya et al. 2023). However, existing health technology assessment processes in Australia uses QALYs as the preferred health outcome measure (Commonwealth of Australia 2024), which informed our decision to include it our study.

Our study excluded other healthcare costs from the base-case scenario analysis due to its relative uncertainty, which would result in less rigour. Therefore, it was included under sensitivity analysis, although it still did not significantly change the probability for intervention cost-effectiveness. Additionally, the exclusion of travel costs for school-based fluoride varnish and school-based dental sealant programs would contribute to an underestimate of the true costs, which would have decreased the probability for their cost-effectiveness in the present analysis. Furthermore, the extrapolation of the intervention effectiveness of the three dental caries preventive interventions under the sensitivity analysis may not be realistic when considered in the real-world scenario.

## Conclusion

Our study demonstrated that anticipatory guidance provided by oral health therapists via home visits and telehealth, school-based fluoride varnish programs provided either by dental practitioners or non-dental health professionals or fissure sealant programs were not cost-effective interventions. The probability of cost-effectiveness increased with a 12-year time horizon, but longer-term effectiveness studies are required to reduce uncertainty to offer definitive findings.

## Electronic supplementary material

Below is the link to the electronic supplementary material.

Supplementary Material 1

Supplementary Material 2

Supplementary Material 3
