# Supplementary material for: Economic evaluation on dental caries preventive interventions for Australian children using a priority-setting approach
Source: Eur J Health Econ. 2025 Apr 30;26(9):1525–37. doi: 10.1007/s10198-025-01787-2 (PMC12618282; doi:10.1007/s10198-025-01787-2)
Supplement: Supplementary file 2 — Supplementary Material 2 [file 10198_2025_1787_MOESM2_ESM.docx]

**Supplementary File**

**Title:** Economic evaluation on dental caries preventive interventions for children using a priority-setting approach in Australia.

**Journal:** PharmacoEconomics

**Authors:** Tan Minh Nguyen, Long Khanh-Dao Le, Hanny Calache, Cathrine Mihalopoulos.

**Correspondence:** Tan Minh Nguyen, Monash University Health Economics Group (MUHEG), School of Public Health & Preventive Medicine, Faculty of Medicine, Nursing and Health Sciences, Monash University; email: tan.nguyen@monash.edu.

**Selection criteria for the Assessing Cost-Effectiveness of Oral Health Preventive Interventions (ACE-Oral Health Prevention)**

1. ***Evidence for effectiveness and strength of evidence*** – considers whether the intervention has demonstrated efficacy based on the strength of evidence.

🗸 = Yes; based on systematic review(s) and meta-analysis and/or multiple studies with randomised controlled trials

? = Neutral; has lower strength of evidence or there is no available evidence

🗴 = No; based on systematic review(s) and meta-analysis and/or multiple studies with randomised controlled trials

1. ***Economic evaluation exist for intervention*** – considers if there is published literature for economic evaluation on the intervention.

🗸 = Yes

🗴 = No

1. ***Intervention implemented/provided routinely in Australia*** – considers whether the intervention is currently implemented or provided routinely in Australia. For some prevention interventions that are clinically based, an assessment was made from the public or private sector.

🗸 = Yes

? = unknown

🗴 = No

1. ***Co-benefits*** – considers whether the interventions would reduce the risk of the development of both caries and periodontitis or the development and worsening of other diseases.

🗸 = Yes

🗴 = No

1. ***Balance*** – considers whether the intervention targets specific ages of the population and whether it targets caries, periodontitis or both.
2. ***Acceptability*** – considers whether the intervention is acceptable from the perspectives of 1) government, 2) industry and 3) the public, based on the published literature wherever possible.

**Table 5** Detailed parameters that were varied from the dental caries model for implementing the 20% sugar tax in Australia [1]. The clinical effectiveness for the interventions were adjusted to one-year probabilities.

| **Variable** | **Mean value**  **(SD)** | **Uncertainty**  **distribution** | **Data source** |  |
| --- | --- | --- | --- | --- |
| Target population | Table 2 | - | Australian Bureau of Statistics, 2022a [2] |  |
| **High risk adjustment for dental caries incidence** | | | |  |
| Deciduous dentition | 1.32 | - | Do and Spencer 2019^3^  Australian Bureau of Statistics, 2022b [4] |  |
| Permanent dentition | 1.40 | - |  |  |
| **Effectiveness parameters** | | | | |
|  |  |  |  |  |
| Probability of dental caries incidence –  Intervention comparator (6 years) | 0.60  (0.05) | Normal | Koh et al., 2015 [5] |  |
| Probability of dental caries incidence –  Intervention 1a (6 years)  Home visits | 0.45  (0.03) |  |  |  |
| Probability of dental caries incidence –  Intervention 1b (6 years)  Telehealth | 0.42  (0.07) |  |  |  |
| Fluoride varnish effectiveness  Intervention 2a and 2b (3 years) | 0.37  (0.07) | Normal | Marinho et al., 2013 [6] |  |
| Fluoride varnish effectiveness  Intervention 2a and 2b (3 years) | 0.43  (0.07) |  |  |  |
| Fissure sealant effectiveness  Intervention 3 (2 years) | 0.88  (0.03) | Normal | Ahovuo-Saloranta et al., 2017 [7] |  |
| **Cost parameters (AUD$)** | | | | |
| Consultation costs –  Home visits  One visit | 23.33 | - | Koh et al., 2015 [5] |  |
| Travel costs –  Home visits  One visit | 12.34  (0.92) | Gamma |  |  |
| Consultation costs –  Telehealth  One visit | 16.04 | - |  |  |
| Dental screening  One visit | 29.25 | - | Department of Veterans’ Affairs, 2020 [8] |  |
| Fluoride varnish  One visit | 36.80 | - |  |  |
| Fissure sealant (four teeth)  One visit | 196.00 | - |  |  |
| Step-down fee for non-dental health professionals providing school-based fluoride varnish program (%) | 0.769 | - | Virginia Health Catalyst 2021 [9] |  |
| **Sensitivity analysis** | | | |  |
| Costs for potentially preventable hospitalisation  Age <7 years old only  Per dental caries case | 14,986 | - | Independent Health and Aged Care Pricing Authority, 2021 [10] |  |
| Costs for general anaesthesia  Age <7 years old only  Per dental caries case | 2,395.44  (183.06) | Gamma | Koh et al., 2015 [5] |  |
| Other healthcare costs | Refer to Other_Healthcare_  Costs_Data_File.xlsx | - | Refer to **Other healthcare costs used for sensitivity analysis** in the Supplementary File. |  |
| **Health outcomes** | | | |  |
| Utility per dental caries case | 0.9  (0.12) | Normal | Koh et al., 2015 [5] |  |
| Utility gained per dental caries case treated | $\frac{1- Utility for caries}{2}$ | Normal |  |  |

SD = standard deviation.

Relevant costs inflated to 2020 prices according to the Reserve Bank of Australia, 2024 [11].

**Other healthcare costs used for sensitivity analysis**

Other healthcare costs were applied for sensitivity analysis. It included the yearly average cost per age-specific individual for common dental services related to the management of dental caries, and weighted according to the mix of private (85) and public (15%) delivery of services.^1^ Refer to ‘Other_Healthcare_Costs_Data_File.xlsx’, spreadsheet ‘Weighted_Private_Public’.

Dental services rates for private and public practice were sourced from Medibank Australia (2019 year), the Australian Government Department of Health and Aged Care (supplied by Services Australia) for the Chronic Disease Dental Scheme (2012 year, program has since ceased), the Child Dental Benefits Schedule (2019 year), and Victorian (2019 year) and Tasmanian (2019 years) state funded dental services.

Additional weightings were applied to account for the likely proportion of the dental service likely attributed to the management of dental caries, which was multiplied by the cost ($AUD) of the dental service in 2020 values. They were determined based on the existing literature, where relevant, and expert opinion as determined by co-authors TMN and HC. Refer to ‘Other_Healthcare_Costs_Data_File.xlsx’, spreadsheet ‘Dental_Service_Weightings_Costs’.

**Table 6** The healthcare cost difference, effectiveness differences, the ICER values and the probability for cost-effectiveness for the three dental caries preventive intervention targeting children at higher risk in Australia (undiscounted).

| **Base-Case Scenario** | **∆ Healthcare**  **Costs* AUD$**  **(SE)** | **∆ DT Effectiveness* (SE)** | **∆ DALY Averted* (SE)** | **∆ QALY Effectiveness* (SE)** | **Incremental Cost-Effectiveness Ratio** | | | | |
| --- | --- | --- | --- | --- | --- | --- | --- | --- | --- |
|  |  |  |  |  | **AUD$ / DT Prevented** | **AUD$ / DALY Averted** | **CE** | **AUD$ / QALY Gained** | **CE** |
| Home Visits | 24.6 million  (<0.1 million) | 8,810  (108) | 0.5  (<0.1) | 2.8  (0.1) | 2,797 | 50.7 x 10^6^ | 0% | 8.8 x 10^6^ | 0% |
| Telehealth | 19.7 million  (<0.1 million) | 10,282  (131) | 0.6  (<0.1) | 3.2  (<0.1) | 1,917 | 34.5 x 10^6^ | 0% | 6.1 x 10^6^ | 0% |
| Dental screening and fluoride varnish | 58.3 million  (0.2 million) | 67,325  (752) | 5.2  (<0.1) | 29.7  (0.6) | 886 | 11.2 x 10^6^ | 0% | 2.0 x 10^6^ | 0% |
| Fluoride varnish  (non-dental health professionals) | 35.2 million  (0.2 million) |  |  |  | 523 | 6.8 x 10^6^ | 0% | 1.2 x 10^6^ | 0% |
| Dental screening and fissure sealant | 10.5 million  (<0.1 million) | 1,928  (22) | 0.1  (<0.1) | 0.8  (<0.1) | 5,444 | 79.2 x 10^6^ | 0% | 13.9 x 10^6^ | 0% |
| **Sensitivity Analysis^**  **Including Other Healthcare Costs** | | | | | | | | | |
| Home Visits | 12.9 million  (<0.1 million) | 8,810  (108) | 0.5  (<0.1) | 2.8  (0.1) | 1,461 | 26.5 x 10^6^ | 0.2% | 4.6 x 10^6^ | 0.2% |
| Telehealth | 5.9 million  (0.1 million) | 10,282  (131) | 0.6  (<0.1) | 3.2  (<0.1) | 577 | 10.4 x 10^6^ | 11.9% | 1.8 x 10^6^ | 11.9% |
| Dental screening and fluoride varnish | 51.3 million  (0.2 million) | 67,325  (752) | 5.2  (<0.1) | 29.7  (0.6) | 762 | 9.9 x 10^6^ | 0% | 1.7 x 10^6^ | 0% |
| Fluoride varnish  (non-dental health professionals) | 28.2 million  (0.2 million) |  |  |  | 419 | 5.4 x 10^6^ | 0.1% | 1.0 x 10^6^ | 0.1% |
| Dental screening and fissure sealant | 10.3 million  (<0.1 million) | 1,928  (22) | 0.1  (<0.1) | 0.8  (<0.1) | 5,337 | 77.6 x 10^6^ | 0% | 12.6 x 10^6^ | 0% |
| **Sensitivity Analysis^**  **12 Year Time Horizon** | | | | | | | | | |
| Home Visits | 8.7 million  (0.1 million) | 22,412  (270) | 1.4  (<0.1) | 7.9  (0.2) | 388 | 6.3 x 10^6^ | 5.9% | 1.1 x 10^6^ | 6.5% |
| Telehealth | 1.2 million  (0.1 million) | 25,837  (323) | 1.6  (<0.1) | 9.0  (0.2) | 45 | 0.7 x 10^6^ | 40.1% | 0.1 x 10^6^ | 41.2% |
| Dental screening and fluoride varnish | -11.5 million  (0.7 million) | 349,627  (3,906) | 48.0  (0.4) | 274.0  (5.4) | -33^#^ | -239,368^#^ | 54.0% | -41,925 | 61.2% |
| Fluoride varnish  (non-dental health professionals) | -37.0 million  (0.7 million) |  |  |  | -106^#^ | -771,546^#^ | 100% | -135,136^#^ | 100% |
| Dental screening and fissure sealant | 1.2 million  (<0.1 million) | 35,912  (401) | 5.2  (<0.1) | 29.5  (0.6) | 34 | 233,107 | 34.8% | 40,803 | 39.0% |
| ^ includes costs of potentially preventable hospitalisation and general anaesthesia for children <7 years old and other healthcare costs related to dental caries;  AUD$ = 2020 Australian dollars; ∆ = change; *0% discount rate was applied; DT = decayed teeth; DALY = disability-adjusted life years; QALY = quality-adjusted life years; CE = probability intervention is cost-effective (AUD$50,000 per DALY averted and AUD$28,033 per QALY gained); ^#^ dominant. | | | | | | | | | |

**Table 7** The implementation considerations for the three dental caries preventive intervention targeting children at higher risk in Australia.

| **Anticipatory Guidance by Oral Health Therapists Home Visits or Telehealth Consultations (Intervention 1a or 1b)** | | | | | |
| --- | --- | --- | --- | --- | --- |
| **Considerations** | | **Details** | | | **Assessment** |
| **Strength of evidence** | | Effectiveness evidence for home visits and telehealth consultations were based on a single case-control Australian study for home visit and telehealth consultations compared to usual care over 5.5 years [5,12].  A similar randomised controlled trial study for home visits in Malaysia demonstrated statistically significant clinical effectiveness for children aged 5-6 years over 2 years [13].  A Cochrane systematic review on diet and feeding practice advice for infants and young children demonstrated statistically significant clinical effectiveness to reduce dental caries prevalence [14]. It included pre- and postnatal visits and home visit interventions by healthcare workers. | | | Medium |
| **Safety** | | The intervention has no safety concerns. | | | High |
| **Acceptability** | **Government** | The intervention was conducted by public dental services in Queensland [5,12]. It is currently not implemented more broadly in Australia. | | | Medium |
|  | **Industry** | Interventions are not routinely provided by oral health therapists in dental practice.  Home visit and telehealth consultations are provided by other health practitioners such as midwives and maternal child health nurses, although the extent of practice is variable between the states and territories. | | | Medium |
|  | **Other stakeholders** | The PSG notes the interventions has likely acceptability by other stakeholders. | | | Positive |
|  | **General public** | The PSG notes the interventions has likely acceptability by the general public. | | | Positive |
| **Equity** | | Home visit and telehealth consultations were implemented and targeted to populations for lower socioeconomic disadvantage [12]. | | | Positive |
| **Feasibility** | | An expanded public oral health workforce is needed to implement and translate home visits and telehealth consultations into practice.  The public oral health workforce is in short supply and has limited government funding to provide public dental services. | | | Low |
| **Sustainability** | | Dental services items can be billed through the state/territory dental programs.  Dental practitioners are typically funded to provide clinical services rather than outreach oral health promotion activities. | | | Low |
| **Environmental impacts** | | Home visits need travel requirements, which produces carbon emissions when travelling by car, although is offset by minimising the need for mothers to travel to the public dental service. | | Reduced travel costs and reduction in dental treatments has an impact on carbon emissions resulting from the provision of oral healthcare [15]. | Neutral |
|  |  | Telehealth consultations do not need travel requirements, although would need information technology systems to support, which may require the purchase of additional equipment. | |  | Positive |
| **Other considerations** | | Home visits and telehealth consultations are likely to support positive behaviour changes that promote oral health to mothers [16,17]. | | | Positive |
| **School-based Dental Screening and Fluoride Varnish Programs**  **(Intervention 2a and 2b) or**  **School-Based Dental Screening and Fissure Sealant Programs (Intervention 3)** | | | | | |
| **Considerations** | | **Details** | | | **Assessment** |
| **Strength of evidence** | | The Cochrane systematic review on school dental screening programs for oral health identified no studies that improved oral health [18].  The Cochrane systematic review and meta-analysis showed the statistical significant difference for the caries preventive effect of 6-monthly fluoride varnish applications [6].  The Cochrane systematic review and meta-analysis showed the statistical significant difference for the caries preventive effect of fissure sealant versus no sealant [7]. | | | High |
| **Safety** | | Common safety concerns for fluoride varnish are likely caused by allergy related incidents rather than toxic doxes of fluoride exposure [6,19]. There has been no documented adverse effects for fluoride or fissure sealant [20].  The 2019 updated Australian fluoride guideline supports the use of fluoride varnish for people with elevated risk of developing caries, including children under the age of 10 years [21].  Fissure sealants is recommended for children and adolescents at higher risk for dental caries [22]. | | | High |
| **Acceptability** | **Government** | Various state/territory led school-based dental program include the use of fluoride varnish and fissure sealant for children at higher risk for dental caries in clinical practice such as in the Northern Territory [23], Tasmania [24], and in Victoria through Smile Squad [25] and other local programs delivered by community dental agencies [26].  Aboriginal dental assistants have been trained in New South Wales to apply fluoride varnish in school settings [27]. | | | High |
|  | **Industry** | Private mobile dental services include the use of fluoride varnish and fissure sealant in clinical practice [28].  Strong support for non-dental health professionals to apply fluoride varnish to high risk populations [29].  Acceptability is likely high for industry suppliers of fluoride varnish and fissure sealants products. | | | High |
|  | **Other stakeholders** | The PSG notes the interventions is likely acceptability by other stakeholders. | | | High |
|  | **General public** | The PSG notes the interventions has likely acceptability by the general public. | | | Positive |
| **Equity** | | | School-based dental programs targeted to school-settings of lower socioeconomic advantage [24–26]. | | Positive |
| **Feasibility** | | | An expanded public oral health workforce is needed to expand school-based dental programs.  The public oral health workforce is in short supply and has limited government funding to provide public dental services. | | Medium |
| **Sustainability** | | | Government funding commitment would be required to maintain continuity of school-based dental programs.  Previous research indicates sustainability achievable using existing funding stream from the Child Dental Benefits Schedule for fluoride varnish programs [30]. | | High |
| **Environmental impacts** | | | School-based dental programs produce carbon emissions when travelling by car by oral healthcare teams, although it is offset by minimising the need for the primary carers to travel to the public dental service.  Reduced travel costs and reduction in dental treatments has an impact on carbon emissions resulting from the provision of oral healthcare [15]. | | Positive |
| **Other considerations** | | | School-based dental programs access to public dental services [31,32]. It is also cost-effective four outcomes related to quality-adjusted tooth years and reaching children from underserved populations [33]. | | Positive |

PSG = project steering group

**References**

1. Nguyen TM, Tonmukayakul U, Khanh-Dao Le L, et al. Modeled health economic and equity impact on dental caries and health outcomes from a 20% sugar sweetened beverages tax in Australia. Health Econ. 2023;32(11):2568-2582. doi:10.1002/hec.4739

2. Australian Bureau of Statistics. National, state and territory population, December 2021. Canberra (AU): Australian Bureau of Statistics (ABS). 2022. https://www.abs.gov.au/statistics/people/population/national-state-and-territory-population/latest-release. Accessed 20 August 2024.

3. Do L, Spencer A. The National Child Oral Health Study 2012–14. Adelaide (AU): University of Adelaide Press. 2016. doi:10.20851/ncohs

4. Australian Bureau of Statistics. Household and families: Census, 2021. Canberra (AU): Australian Bureau of Statistics (ABS). 2022. https://www.abs.gov.au/statistics/people/people-and-communities/household-and-families-census/latest-releasehttps://www.abs.gov.au/statistics/people/people-and-communities/household-and-families-census/2021/Household%20and%20families%20data%20summary.xlsx. Accessed 20 August 2024.

5. Koh R, Pukallus M, Kularatna S, et al. Relative cost-effectiveness of home visits and telephone contacts in preventing early childhood caries. Community Dent Oral Epidemiol. 2015;43(6):560-568. doi:10.1111/cdoe.12181

6. Ahovuo-Saloranta A, Forss H, Walsh T, Nordblad A, Mäkelä M, Worthington HV. Pit and fissure sealants for preventing dental decay in permanent teeth. Cochrane Oral Health Group, ed. Cochrane Database Syst Rev. 2017;2017(7). doi:10.1002/14651858.CD001830.pub5

7. Ahovuo-Saloranta A, Forss H, Walsh T, Nordblad A, Mäkelä M, Worthington HV. Pit and fissure sealants for preventing dental decay in permanent teeth. Cochrane Oral Health Group, ed. Cochrane Database of Systematic Reviews. 2017;2017(7). doi:10.1002/14651858.CD001830.pub5

8. Department of Veterans’ Affairs. Dental and allied health fee schedules. Canberra (AU): Australian Government. 2020. https://www.dva.gov.au/get-support/providers/fees-forms-claims-providers/fee-schedules/dental-and-allied-health-fee-schedules. Accessed 20 August 2024.

9. Virginia Health Catalyst. Fluoride Varnish. 2021. https://vahealthcatalyst.org/provider-resources/fluoride-varnish/. Accessed 20 August 2024.

10. Independent Health and Aged Care Pricing Authority. NWAU calculators. Canberra (AU): Independent Health and Aged Care Pricing Authority (IHACPA). 2021. https://www.ihacpa.gov.au/health-care/pricing/nwau-calculators. Accessed 20 August 2024.

11. Reserve Bank of Australia. Inflation Calculator. Canberra (AU): Reserve Bank of Australia (RBA). 2024. https://www.rba.gov.au/calculator. Accessed 20 August 2024.

12. Plonka KA, Pukallus ML, Barnett A, Holcombe TF, Walsh LJ, Seow WK. A controlled, longitudinal study of home visits compared to telephone contacts to prevent early childhood caries. Int J Paediatr Dent. 2013;23(1):23-31. doi:10.1111/j.1365-263X.2011.01219.x

13. Babar MG, Andiesta NS, Bilal S, Yusof ZYM, Doss JG, Pau A. A randomized controlled trial of 6‐month dental home visits on 24‐month caries incidence in preschool children. Comm Dent Oral Epid. 2022;50(6):559-569. doi:10.1111/cdoe.12710

14. Riggs E, Kilpatrick N, Slack-Smith L, et al. Interventions with pregnant women, new mothers and other primary caregivers for preventing early childhood caries. Cochrane Database Syst Rev. 2019;2019(11):CD012155. doi:10.1002/14651858.CD012155.pub2

15. Duane B, Harford S, Ramasubbu D, et al. Environmentally sustainable dentistry: a brief introduction to sustainable concepts within the dental practice. Br Dent J. 2019;226(4):292-295. doi:10.1038/s41415-019-0010-7

16. Tannous KW, George A, Ahmed MU, et al. Economic evaluation of the Midwifery Initiated Oral Health-Dental Service programme in Australia. BMJ Open. 2021;11(8):e047072. doi:10.1136/bmjopen-2020-047072

17. Rana K, Ekanayake K, Chimoriya R, et al. Effectiveness of Oral Health Promotion Interventions: An Evidence Check Rapid Review Brokered by the Sax Institute and Commissioned by Dental Health Services Victoria for the Victorian Department of Health. The Sax Institute; 2022. doi:10.57022/oiik8302

18. Arora A, Kumbargere Nagraj S, Khattri S, Ismail NM, Eachempati P. School dental screening programmes for oral health. Cochrane Database Syst Rev. 2022;7(7):CD012595. Published 2022 Jul 27. doi:10.1002/14651858.CD012595.pub419. Mascarenhas AK. Is fluoride varnish safe?: Validating the safety of fluoride varnish. J Am Dent Assoc. 2021;152(5):364-368. doi:10.1016/j.adaj.2021.01.013

20. Papageorgiou SN, Dimitraki D, Kotsanos N, Bekes K, van Waes H. Performance of pit and fissure sealants according to tooth characteristics: A systematic review and meta-analysis. J Dent. 2017;66:8-17. doi:10.1016/j.jdent.2017.08.004

21. Do L, Australian Research Centre for Population Oral Health. Guidelines for use of fluorides in Australia: update 2019. Aust Dent J. 2020;65(1):30-38. doi:10.1111/adj.12742

22. Wright JT, Crall JJ, Fontana M, et al. Evidence-based clinical practice guideline for the use of pit-and-fissure sealants. J Am Dent Assoc. 2016;147(8):672-682.e12. doi:10.1016/j.adaj.2016.06.001

23. Australian Institute of Health and Welfare. Northern Territory Remote Aboriginal Investment: Oral Health Program July 2012 to December 2017. Cat no. IHW 205. 2019.

24. Tasmanian Department of Health. Fissure sealant and fluoride varnish program. Hobart (AU): Tasmanian Department of Health. 2021. https://www.health.tas.gov.au/health-topics/dental-health/dental-health-programs-and-initiatives/fissure-sealant-and-fluoride-varnish-program. Accessed 20 August 2024.

25. Victorian Department of Health. Smile Squad services. 2024. Melbourne (AU): State Government of Victoria. https://www.health.vic.gov.au/smile-squad-services. Accessed 20 August 2024.

26. Mason A, Mayze L, Pawlak J, Henry MJ, Sharp S, Smith MC. A Preventative Approach to Oral Health for Children in a Regional/Rural Community in South-West Victoria, Australia. Dentistry. 2015;05(07). doi:10.4172/2161-1122.1000313

27. Skinner J, Dimitropoulos Y, Masoe A, et al. Aboriginal dental assistants can safely apply fluoride varnish in regional, rural and remote primary schools in New South Wales, Australia. Australian J Rural Health. 2020;28(5):500-505. doi:10.1111/ajr.12657

28. Nguyen TM, Tonmukayakul U, Calache H. A Cost Analysis of an Outreach School-Based Dental Program: Teeth on Wheels. Children (Basel). 2021;8(2):154. Published 2021 Feb 18. doi:10.3390/children8020154

29. Skinner J, Dimitropoulos Y, Sohn W, et al. Child Fluoride Varnish Programs Implementation: A Consensus Workshop and Actions to Increase Scale-Up in Australia. Healthcare. 2021;9(8):1029. doi:10.3390/healthcare9081029

30. Skinner J, Dimitropoulos Y, Rambaldini B, et al. Costing the Scale-Up of a National Primary School-Based Fluoride Varnish Program for Aboriginal Children Using Dental Assistants in Australia. IJERPH. 2020;17(23):8774. doi:10.3390/ijerph17238774

31. Nguyen TM, Morgan M, Koshy S, Mathew S, Lew S. Revisiting the value of school-based dental check-up programs. Aust N Z J Dent Oral Health Ther. 2015; (2):6-12.

32. Nguyen TM, Christian B, Koshy S, Morgan MV. A Validation and Cost-Analysis Study of a Targeted School-Based Dental Check-Up Intervention: Children’s Dental Program. Children (Basel). 2020;7(12). doi:10.3390/children7120257

33. Nguyen TM, Hsueh YS, Morgan MV, Mariño RJ, Koshy S. Economic Evaluation of a Pilot School–Based Dental Checkup Program. JDR Clin Transl Res. 2017;2(3):214-222. doi:10.1177/2380084417708549
