# Supplementary material for: Economic evaluation on dental caries preventive interventions for Australian children using a priority-setting approach
Source: Eur J Health Econ. 2025 Apr 30;26(9):1525–37. doi: 10.1007/s10198-025-01787-2 (PMC12618282; doi:10.1007/s10198-025-01787-2)
Supplement: Supplementary file 3 — Supplementary Material 3 [file 10198_2025_1787_MOESM3_ESM.docx]

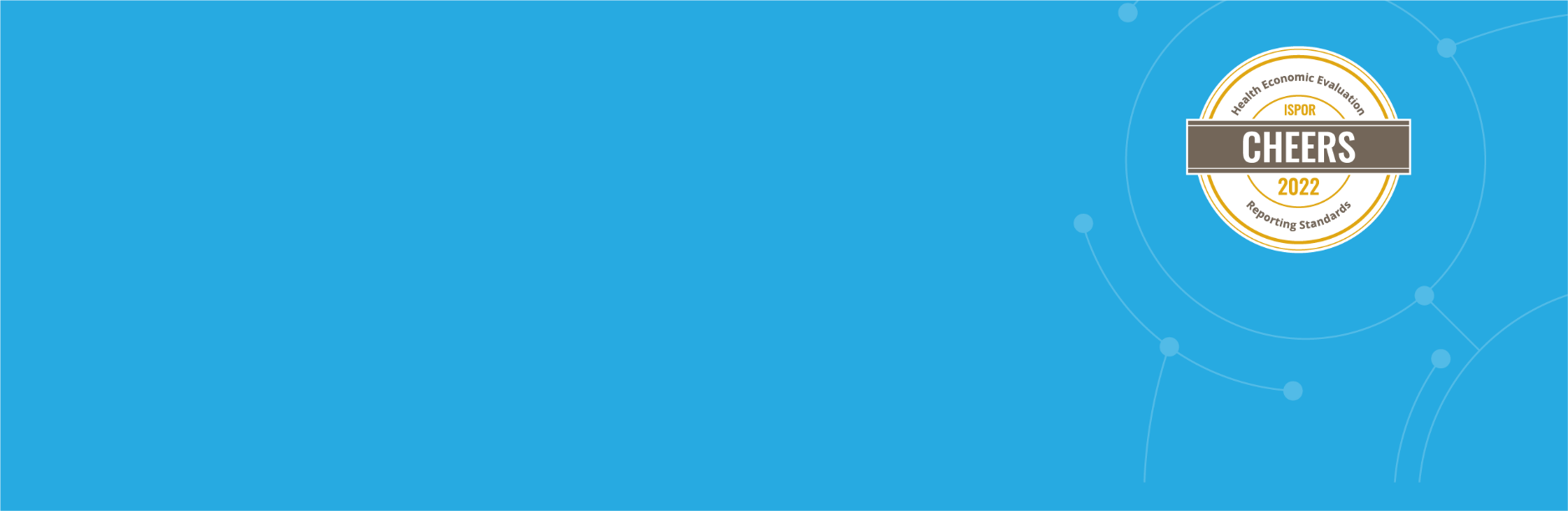


**Consolidated Health Economic Evaluation Reporting Standards
(CHEERS) 2022 Checklist**

The CHEERS 2022 statement replaces the 2013 CHEERS statement, which should no longer
be used. The CHEERS 2022 checklist contains 28 items with accompanying descriptions.
Checklist users should indicate the section of the manuscript where relevant information
can be found. The authors recommend using a section heading with a paragraph number.
If an item does not apply to a particular economic evaluation, checklist users are encouraged
to report “Not Applicable.” If information is otherwise not reported, checklist users are encouraged to

write, “Not Reported.” Users should avoid the term “Not Conducted” as CHEERS is intended to guide and

capture reporting. Additional information on CHEERS 2022 can be found here.

**Title**

**1. Title**

Identify the study as an economic evaluation and specify the interventions being compared.

"Economic evaluation on dental caries preventive interventions for children using a priority-setting approach in Australia".

**Abstract**

**2. Abstract**

Provide a structured summary that highlights context, key methods, results, and alternative analyses.

Included under subheadings: Objective, Methods, Results and Conclusions.

**Introduction**

**3. Introduction: Background and Objectives**

Give the context for the study, the study question, and its practical relevance for decision making in policy or practice.

Study aim were described as follows: "The aim of this study is to undertake an economic evaluation of preventive interventions for dental caries targeting Australian children using a population health approach. The three oral health preventive interventions to be evaluated target children from families with low household income and include: 1) anticipatory guidance provided by oral health therapists, 2) school-based fluoride varnish program, and 3) school-based fissure sealant program."

**Methods**

**4. Health economic analysis plan**

Indicate whether a health economic analysis plan was developed and where available.

Not reported.

**5. Study population**

Describe characteristics of the study population (such as age range, demographics, socioeconomic, or clinical characteristics).

Under subheading "Population" and Table 2.

**6. Setting and location**

Provide relevant contextual information that may influence findings.

Under subheadings "Anticipatory guidance provided by oral health therapists", "School-based fluoride varnish program", "School-based fissure sealant program", and "Other intervention components for school-based programs".

**7. Comparators**

Describe the interventions or strategies being compared and why chosen.

Under subheadings "Effectiveness of dental caries preventive interventions", "Anticipatory guidance provided by oral health therapists", "School-based fluoride varnish program", "School-based fissure sealant program", and "Other intervention components for school-based programs".

**8. Perspective**

State the perspective(s) adopted by the study and why chosen.

"The Australian government healthcare perspective (intervention and dental caries treatment costs) was adopted in this study given these interventions are reliant on the public healthcare system for implementation."

**9. Time horizon**

State the time horizon for the study and why appropriate.

"Two separate time horizons in the base case (Table 2) were selected to reflect the duration on the evidence of effectiveness for the dental caries preventive interventions: 1) six years for anticipatory guidance in early childhood delivered by oral health therapists and 2) two years for school-based dental screening with fluoride varnish and school-based fissure sealant program."

**10. Discount rate**

Report the discount rate(s) and reason chosen.

"A discount rate of 3% was applied for both costs and outcome measures, consistent with the previous published dental caries model."

**11. Selection of outcomes**

Describe what outcomes were used as the measure(s) of benefit(s) and harm(s).

Under subheading "Health benefit modelling".

**12. Measurement of outcomes**

Describe how outcomes used to capture benefit(s) and harm(s) were measured.

Under subheading "Health benefit modelling".

**13. Valuation of outcomes**

Describe the population and methods used to measure and value outcomes.

Not applicable.

**14. Measurement and valuation of resources and costs**

Describe how costs were valued.

Under subheading "Intervention costs", "Healthcare costs" and "Other healthcare costs".

**15. Currency, price date, and conversion**

Report the dates of the estimated resource quantities and unit costs, plus the currency and year of conversion.

"All costs were adjusted to 2020 (AUD$) prices based on the consumer price index/"

**16. Rationale and description of model**

If modeling is used, describe in detail and why used. Report if the model is publicly available and where it can be accessed.

"For this study, the previously published dental caries economic model from the 20% sugar-sweetened beverages tax intervention was adapted."

**17. Analytics and assumptions**

Describe any methods for analyzing or statistically transforming data, any extrapolation methods, and approaches for validating any model used.

Under subheadings "Extrapolation modelling" and "Model validation". Also reported in Supplementary File.

**18. Characterizing heterogeneity**

Describe any methods used for estimating how the results of the study vary for subgroups.

Not applicable.

**19. Characterizing distributional effects**

Describe how impacts are distributed across different individuals or adjustments made to reflect priority populations.

Not applicable.

**20. Characterizing uncertainty**

Describe methods to characterize any sources of uncertainty in the analysis.

"Monte Carlo simulation with 2,000 cycles using within cycle correction was performed in TreeAge Pro 2023 to conduct probabilistic sensitivity analysis. Summary statistics and the incremental cost-effectiveness ratio (ICER) were analysed using Stata 18 BE (StataCorp). Key parameters varied in the model structure were the start age, number of children in the single age cohorts, intervention effectiveness and intervention cost parameters (Supplementary File, Table 5)."

**21. Approach to engagement with patients and others affected by the study**

Describe any approaches to engage patients or service recipients, the general public, communities, or stakeholders (eg, clinicians or payers) in the design of the study.

"Acknowledgements: The authors would like to thank members of the Project Steering Group for ACE-Oral Health Prevention for this study Dr Clare Lin (Dental Health Services Victoria), Mr Rick Monty (Representative, National Dental Directors), Mr Tony McBride (Spokesperson, Victorian Oral Health Alliance), Ms Bree Jones (Australian Dental and Oral Health Therapists’ Association), Mr Robin Brown (Consumer Representative), and Prof John Skinner (Health Research Institute). Additional appreciation for Dr Utsana Tonmukayakul (Deakin University) in contributing to the modelling approach. Additionally, the authors would like to thank dental service rate data supplied by the Medibank Private, Services Australia, Victorian Department of Health/Dental Health Services Victoria, and the Tasmanian Department of Health".

Under subheading "Methods": "Based on our own evidence review on the effectiveness of different preventive interventions for oral health, the three preventive interventions investigated in this study were selected by the Project Steering Group."

**Results**

**22. Study parameters**

Report all analytic inputs (eg, values, ranges, references) including uncertainty or distributional assumptions.

Under suheading "Methods" and Table 1, 2, and Supplementary File.

**23. Summary of main results**

Report the mean values for the main categories of costs and outcomes of interest and summarize them in the most appropriate overall measure.

Subheading "Results" and Table 3, 4.

**24. Effect of uncertainty**

Describe how uncertainty about analytic judgments, inputs, or projections affects findings. Report the effect of choice of discount rate and time horizon, if applicable.

Under subheading "Results" and Table 3, 4, and Supplementary File.

**25. Effect of engagement with patients and others affected by the study**

Report on any difference patient/service recipient, general public, community, or stakeholder involvement made to the approach or findings of the study.

Not repprted.

**Discussion**

**26. Study findings, limitations, generalizability, and current knowledge**

Report key findings, limitations, ethical, or equity considerations not captured and how these could impact patients, policy, or practice.

Under subheading "Discussion" and Table 4.

**Other Relevant Information**

**27. Source of funding**

Describe how the study was funded and any role of the funder in the identification, design, conduct, and reporting of the analysis.

"Funding/Support: This study was supported by the National Health and Medical Research Council (NHMRC) Postgraduate Scholarship Scheme (APP1189802) and the Australian Government Research Training Program (RTP) Scholarship. LL, HC and CM received no financial support for this research."

**28. Conflicts of interest**

Report authors’ conflicts of interest according to journal or International Committee of Medical Journal Editors requirements.

"Conflicts of Interest: Mr Nguyen reports grants from the NHMRC Postgraduate Scholarship Scheme (APP1189800), Drs Le, Calache, and Mihalopoulos has nothing to disclose."
